# Analgesic effects of nerve growth factor‐directed monoclonal antibody on diabetic neuralgia in an animal model

**DOI:** 10.1002/2211-5463.13410

**Published:** 2022-05-03

**Authors:** Xingchen Dong, Huanhuan Li, Min Pei, Jie Tan, Ganjun Chen, Santai Li, Zuobin Xie, Qi Wang, Guifeng Wang, Yi‐Li Chen, Chunhe Wang

**Affiliations:** ^1^ School of Chinese Materia Medica Nanjing University of Chinese Medicine Nanjing Jiangsu China; ^2^ Shanghai Mabstone Biotechnology, Ltd Shanghai China; ^3^ Biotherapeutics Discovery Research Center Shanghai Institute of Materia Medica, Chinese Academy of Sciences Shanghai China; ^4^ Dartsbio Pharmaceuticals, Ltd Zhongshan Guangdong China

**Keywords:** diabetic neuralgia, neuropathic pain, nerve growth factor, monoclonal antibody, analgesia

## Abstract

Current treatment options for diabetic neuralgia are limited and unsatisfactory. Tanezumab, a monoclonal antibody that blocks nerve growth factor (NGF) signaling, has been shown to be effective in relieving the clinical symptoms of osteoarthritis pain, chronic low back pain, cancer pain induced by bone metastasis, and diabetic neuralgia. However, the clinical development of tanezumab has been terminated due to the risk of induction of rapidly progressive osteoarthritis (RPOA), and no other NGF antibodies have been examined for their ability to treat diabetic neuralgia in either animal models or clinical trials. In this study, a humanized high‐affinity NGF monoclonal antibody (mAb), huAb45 that could neutralize the interaction between NGF and its high‐affinity receptor TrkA. In a mouse diabetic neuralgia model, it effectively relieved neuropathic pain. This study may serve as the necessary foundation for future studies of huAb45 to potentially treat diabetic neuralgia.

## Graphical abstract

NGF‐directed monoclonal antibody blocks the binding of NGF to its receptor, TrkA. Subsequently this blocks downstream signaling that interrupts diabetic neuralgia signaling.

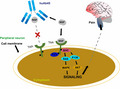

**Abbreviations**
mAb: monoclonal antibody
NGF: nerve growth factor
NSAIDs: nonsteroidal antiinflammatory drugs
RPOA: rapidly progressive osteoarthritis
TrkA: tropomyosin‐associated kinase A

Diabetic neuropathy is one of the most common and dangerous diabetic complications that are difficult to treat [1]. The exact cause of diabetic neuralgia is unclear, possibly due to metabolic imbalance induced by hyperglycemia, neurovascular ischemia and hypoxia, decreases in neurotrophic factors, or the combined effect of autoimmune factors [2]. Currently, the only clinically proven strategy is to manage the blood sugar in combination with analgesics, such as amitriptyline, gabapentin, and pregabalin [3]. Gabapentin is a drug commonly used in the treatment of neuralgia, and its analgesic effect is mostly associated with an auxiliary α2δ subunit of a voltage‐gated calcium channel. When α2δ is bound with gabapentin, the membrane and axoplasmic transport will be inhibited, and neurotransmitter release will decrease, resulting in antiepileptic and antineuralgia effects [4, 5, 6]. The most common side effects of these drugs are tiredness, dizziness, and gastrointestinal responses, which are more common in the elderly and frail, sensitive individuals, and patients with peripheral edema [7, 8]. In addition, metabolically and nutritionally these drugs can cause increased blood sugar and weight gain [9, 10]. Long‐term use is not recommended due to the above‐mentioned side effects of these drugs and their tendency to induce resistance. Therefore, there is an urgent need for therapies that may effectively relieve diabetic neuralgia with minimal side effects.

Nerve growth factor (NGF) is a neurotrophic factor that regulates the growth and development of peripheral and central neurons, maintains neuronal activities, and is involved in pain perception and processing [11]. There are two different receptors for NGF: tropomyosin‐associated kinase A (TrkA) and tumor necrosis factor receptor superfamily member p75NTR [12]. Both receptors dimerize in response to NGF binding. Homodimers of TrkA and heterodimers of TrkA and p75NTR promote cell survival, while p75NTR homodimers mediate apoptosis. Additionally, elevation of NGF expression levels has been identified in many clinical disorders, including rheumatoid arthritis, osteoarthritis, spondyloarthropathies, and lumbar degenerative disk disease. Thus, the inhibition of NGF/TrkA signaling may effectively reduce pain with limited side effects [13]. Pharmacological therapies, targeting the NGF/TrkA signaling pathway, especially mAb, represent a type of promising nonaddictive analgesic with efficacy comparable to opioids [14]. Tanezumab, the first NGF mAb to reach the clinical stage, showed an excellent character and efficacy in the treatment of diabetic neuralgia in a Phase II clinical trial [15, 16]. Regrettably, this trial was terminated by the developers due to tanezumab’s risk of triggering rapidly progressive osteoarthritis (PROA) in osteoarthritis patients and the imminent expiry of its patent protection [17]. The reason for RPOA is not yet clear, but studies have shown that epitopes are a key factor in the negative effects of antibodies [18]. The antibody obtained in this study has different epitopes from tanezumab, and there is a reason to believe that it will behave differently [19]. Furthermore, there are no other NGF antibodies tested on diabetic neuralgia, clinically or in any animal models [20].

In this study, a humanized NGF mAb, huAb45 that blocks the interaction between NGF and TrkA was generated, and has different epitopes from tanezumab. The epitope, purity, binding and blocking activities, and stability of huAb45 were confirmed *in vitro*. In a mouse model of diabetic neuralgia, huAb45 effectively relieved neuropathic pain. This study may lay the necessary foundation for clinically testing huAb45 on diabetic neuralgia.

## Materials and methods

### Cell lines and reagents

TF‐1 cells were purchased from American Tissue Culture Collection (ATCC, Rockville, MD, USA), and were cultured with RPMI1640 (Gibco, Waltham, MA, USA) supplemented with 10% fetal calf serum (Thermo Fisher Scientific, Waltham, MA, USA) at 37 °C and 5% CO_2_. The Cell Counting Kit‐8 (CCK8) was purchased from Dojindo Laboratories (Tokyo, Japan). Human antibody germline sequences and primers were synthesized by Sangon Biotech (Shanghai, China). Tetramethylbenzidine (TMB) substrate was purchased from Thermo Fisher Scientific, PEG400 and thymidine (HAT) were from Sigma‐Aldrich. The human NGF sequences was fused into 6xHis‐tag and cloned into the pCAT1.0 vector (constructed in Darsbio Ltd.), followed by transient transfection of HEK293 cells for protein production. Amino acid sequences of tanezumab light and heavy chains were obtained from the international Immunogenetics information system (IMGT/mAb‐DB), and huAb45 were generated in‐house as described below.

### Generation of HuAb45

NGF antibodies were generated by the hybridoma technique. BALB/c mice (Beijing Vital River Laboratory Animal Technology Co. Ltd., Beijing, China) were immunized by NGF‐6xHis and keyhole limpet hemocyanin. To form hybridomas, immunized mouse spleen cells were fused with myeloma cell SP2/0 at a 5:1 ratio using PEG400 (Sigma‐Aldrich, Darmstadt, Germany) and grown in 100 mm hypoxanthine, 0.4 mm aminopterin, and 1.6 mm thymidine (HAT) (Sigma‐Aldrich,) selection medium. Enzyme‐linked immunoassay (ELISA) was used to test positive hybridoma cells 12 days after infusion, and then the cells were continually subcloned at least three times with limiting dilution to ensure clonality. Hybridoma cells capable of producing high‐sensitivity monoclonal antibodies against NGF in cell culture supernatants were recognized by ELISA. HuAb45 used in this study was screened out from a pool of neutralizing antibodies as the most potent blocker of NGF binding to TrkA. The variable regions of heavy (VH) and light (VL) chains were cloned into the human IgG2 backbone by “framework shuffling” technology, and then transiently expressed in HEK293F cells.

### Binding ELISA

ELISA  was used to measure the binding affinity of NGF monoclonal antibodies with antigens derived from different species.

NGF‐6xHis (2 µg·mL^−1^) was coated onto immuno‐plates (Greiner Bio‐One, Monroe, NC, USA) at 4 °C overnight. Antibodies were gradiently diluted to three fold series from 100 nm. After 1 h of incubation, horseradish peroxidase (HRP)‐conjugated goat antihuman IgG (H + L) was added and then incubated for another hour at 37 °C. Next, the plate was washed six times with 0.05% phosphate‐buffered saline tween (PBST) and developed with TMB substrate. The SpectraMax M5e microplate reader (Molecular Devices, Sunnyvale, CA, USA) was used to measure the absorbance of OD_450_.

### Biolayer interferometry (BLI)

BLI was also used to measure the binding affinity of antibodies with antigens derived from different species. The affinities of huAb45 and tanezumab were determined by Fortebio Octet Red 96. All measurements were performed in the sample buffer at room temperature (RT). Antibodies (10 µg·mL^−1^) were immobilized onto Protein A biosensors (Port Washington, NY, USA), and antigens were serially diluted two folds from 100 nm. *KD* values were calculated by the vendor‐supplied software.

### Blocking ELISA

The ELISA assay was used to investigate the ability of huAb45 to block the NGF binding to TrkA. First, TrkA‐Fc (2 μg·mL^−1^) was coated onto 96‐well immune plates at 4 °C overnight. Then, three fold serial dilutions of huAb45 and tanezumab were added into each well for 1 h at room temperature. Next, NGF‐6xHis‐biotin (0.5 μg·mL^−1^) was added into wells for 30 min at room temperature. Finally, the HRP‐conjugated streptavidin was used and the plate was developed with TMB substrate to detect the bounded competitive ligands. The IC_50_ of antibodies was measured by software on a SpectraMax M5e microplate reader.

### Epitope binning

Epitope binning assay was performed on Octet RED96. First, tanezumab and huAb45 (100 nm) were loaded onto protein A sensors as the first antibody, followed by washing in 0.05% PBST to reach a baseline. Second, sensors were exposed to 100 nm of human NGF‐6xHis at the binding step, followed by exposure to the second antibody (100 nm) at the association step. Forte Bio’s Data Analysis Software 6.0 was used to process the data.

### Inhibition of human NGF binding to TF‐1 cells

TF‐1 cells were seeded into 96‐well cell culture plates at 15,000 cells/well. The antibodies to be tested were diluted with 10% FBS in RPMI‐1640, starting from 450 ng·mL^−1^, with 1.4‐fold gradient, 11 gradients, 20 μL/well, and incubated with cells for 30 min in a mixed incubator. NGF‐6xHis was diluted to 25 ng·mL^−1^ with 10% FBS in RPMI‐1640, added into the plate (20 μL/well), and tested after 72 h in the incubator. Cells were finally counted by the CCK8 kit.

### Analgesic effects evaluation in inflammatory mouse models

Osteoarthritis in the arthritis model was induced by sub‐plantar injection of complete Freund’s adjuvant (at the dose of 400 µL every 3 days for 3 weeks) into the left hind paw of 6–8 week‐age male BALB/c mice (Gempharmatech, Jiangsu, China). Three mice were fed in each cage.

On the day of the test, 15–30 min before the measurement, the mice were kept in a glass test cage to make them relaxed and stay still.

A thermal plantar/Tail Flick instrument (IITC, Woodland Hills, CA, USA) was used to place the infrared light source directly below the middle surface of the mouse's hind paw, and then the start button was pressed. The plantar surface of the hind paw was exposed to the beam of radiant heat through the transparent glass surface [21].

The withdrawal latency of injured and uninjured hind paws was recorded in a balanced sequence. The time (sec) from the start of the thermal stimulus to the paw withdrawal from the heat source was automatically recorded. If the answer was ambiguous, the stimulus was repeated, with the latency to be recorded again. Mice shaking or licking hind paws was also a sign of painful irritation.

The thermal stimulus was repeated at least three times at approximately 5‐min intervals, with the latencies recorded, then the average paw withdrawal latencies were calculated.

### Analgesic effects evaluation of HuAb45 on db/db mouse models

To determine the analgesic ability of huAb45, 6–8 week‐age male db/db (Gempharmatech, Jiangsu, China) mouse models, free of neurodegeneration, were used [22]. Three mice were fed in each cage. Gabapentin was used as a positive drug. A “Von Frey filament” was used to measure the pain threshold. Mice from the normal and model groups were subcutaneously injected with the test drug vehicle once a week; mice from the positive control group were given gabapentin (100 mg·kg^−1^) by oral gavage every day for 28 consecutive days. Mice from the test drug groups were injected subcutaneously with the corresponding doses of huAb45 on Day 1 (D1), D8, D15, and D 22, for the first, second, third, and fourth administration, respectively. The 4‐h fasting blood glucose of mice was measured on D6, D13, D20, and D27 of the test, and mice were weighed before fasting. The mechanical pain threshold was measured on D7, D14, D21, and D28 of the test. Before pain measurement, all mice were kept in the test cage to adapt to the environment. A series of von Frey filaments (0.16, 0.4, 0.6, 1.0, 1.4, 2.0, and 4.0 g) were used to measure the foot retraction reaction of mice by the up–down method: the middle of the hind paw in each mouse was vertically stimulated with von Frey filaments to make it slightly S‐shaped for 5 s, with observation of the claw withdrawal reaction [23]. Rapid withdrawal, leg lifting, or foot licking immediately after stimulus were considered a positive reaction; so was immediate withdrawal after removal of the filament; no reaction after filament stimulus was regarded as a negative reaction. The interval between the two measurements was about 10 s. The measurement started with a 0.4 g fiber filament, in case of a withdrawal reaction, recorded as X, and the filament would be replaced with a 0.16 g weaker primary fiber filament; in case of no withdrawal reaction, the filament would be replaced with a 0.6 g stronger primary fiber. After that, the test continued in the same way.

### Animal housing and handling

Mice were fed a standard diet and water and kept in a controlled air‐conditioned environment at 22 ± 2 °C with a relative humidity of 50% ± 10% on a 12‐h/12‐h light/dark cycle. The experiments were performed according to the guidelines outlined by the Institutional Animal Care and Use Committee of the Shanghai Institute of Materia Medica, Chinese Academic Science (Shanghai, China) (IACUC: 2021‐03‐WCH‐05).

## Results

### Generation and characterization of HuAb45

**Fig. 1:**
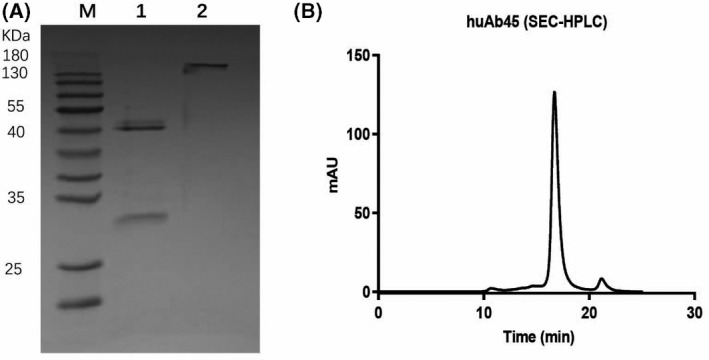
Purity of huAb45 monoclonal antibody. (A) Analysis of huAb45 by reduced and nonreduced SDS‐PAGE. Lane 1: reduced huAb45; Lane 2: nonreduced huAb45; M: molecular weight markers. (B) The purity of huAb45 analyzed by SEC‐HPLC.

**Fig. 2:**
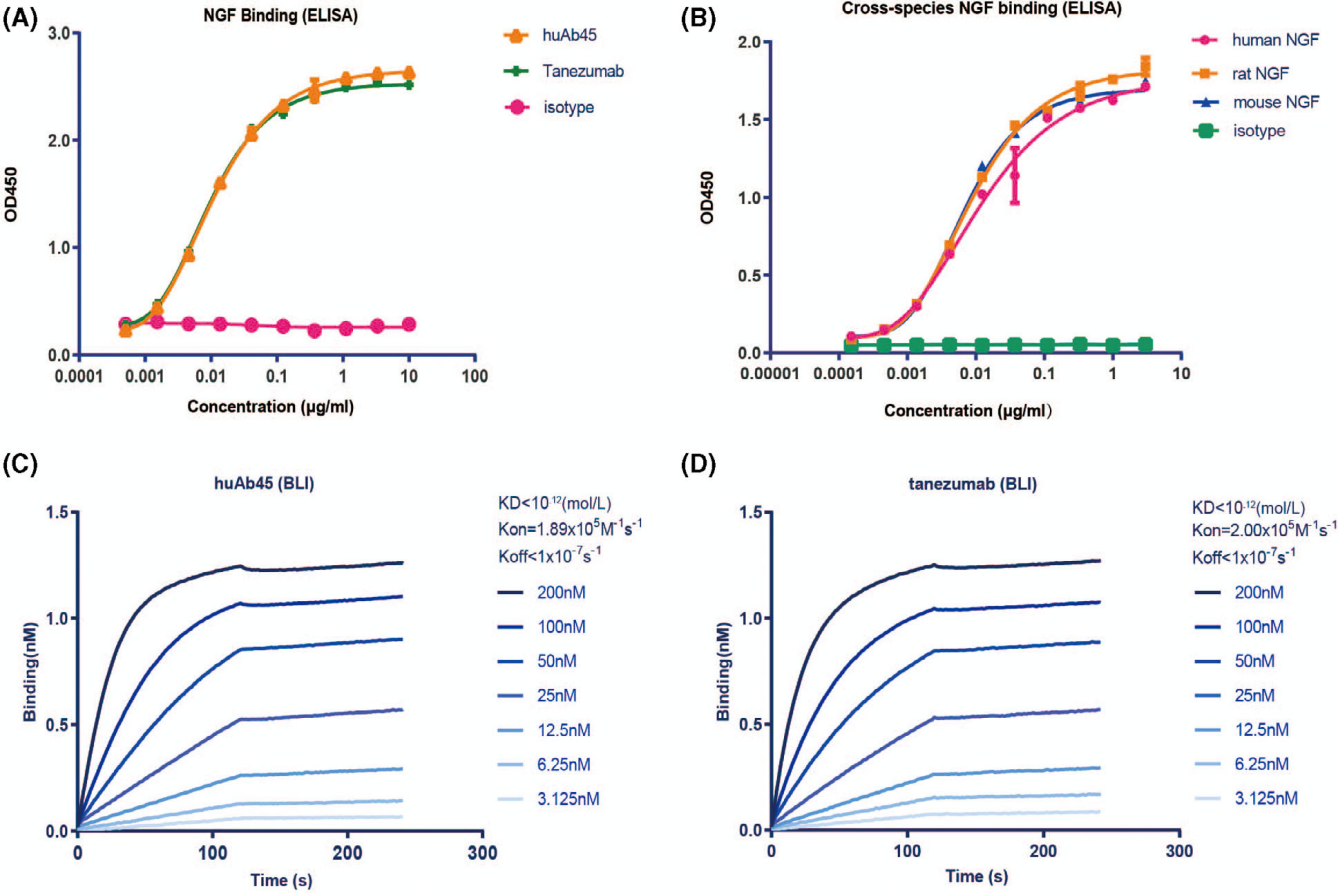
Binding activities of NGF monoclonal antibodies to NGF. (A) Binding activities of huAb45 and tanezumab to 6xHis‐tagged human NGF as measured by ELISA. (B) Binding activities of huAb45 to mouse, rat, and human NGF by ELISA. (C) Binding of huAb45 to 6xHis‐tagged human NGF by BLI. (D) Binding of tanezumab to 6xHis‐tagged human NGF by BLI. Data shown represent *n* = 3. *EC_50_* values were calculated by GraphPad PRISM7.0 (San Diego, CA, USA). Error bars represent SD.

A large number of NGF‐reactive antibodies were cloned from hybridomas established in NGF‐immunized BALB/c mice. It has been demonstrated that a subset of these antibodies can neutralize NGF and TrkA interaction. HuAb45 was humanized and expressed in HEK293 cells. After purification, huAb45 exhibited great purity when characterized by Sodium Dodecyl Sulfate and Polyacrylamide Gel Electrophoresis (SDS‐PAGE) and size exclusion chromatography (SEC‐HPLC) (Fig. 1A,B). Then we compared the binding affinities of huAb45 and tanezumab to 6xHis‐tagged human NGF by ELISA and BLI. *EC_50_* values of huAb45 and tanezumab were 0.01129 and 0.00934 nm by ELISA, respectively (Fig. 2A), and their apparent dissociation constant (KD) values were both less than 1.0E‐12 nm by BLI; human NGF was used as the soluble analyte (Fig. 2C,D). In addition, we evaluated the binding properties of huAb45 to human, mouse, and rat NGF. HuAb45 displayed dose‐dependent binding to human, mouse, and rat NGF (Fig. 2B).

### Neutralizing activities *in vitro*

**Fig. 3:**
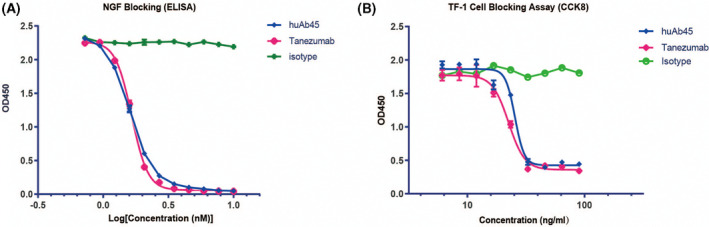
Neutralizing abilities of NGF monoclonal antibody (huAb45). (A) HuAb45 blocked the interaction between NGF and TrkA as measured by ELISA. Tanezumab and isotype were used as positive and negative controls, respectively. (B) HuAb45 suppressed NGF‐dependent TF‐1 cell proliferation detected by CCK‐8. Data shown represent *n* = 3. *IC_50_* values were calculated by GraphPad PRISM7.0. Error bars represent SD.

The binding of NGF to TrkA‐Fc could be effectively blocked by huAb45 and tanezumab measured by ELISA, with IC_50_ values of 1.626 and 1.671 nm, respectively (Fig. 3A). More important, both huAb45 and tanezumab reduced NGF‐induced proliferation of TF‐1 cells. Therefore, huAb45 was a potent neutralizer of NGF signaling *in vitro* (Fig. 3B).

### HuAb45 displayed excellent photostability and thermostability

**Fig. 4:**
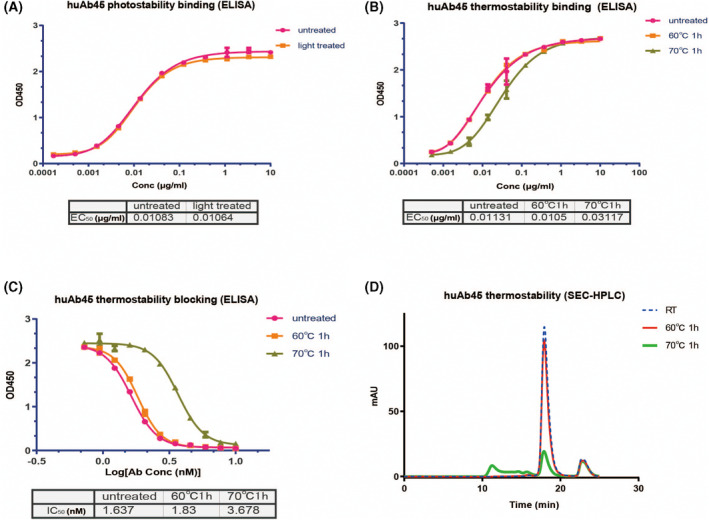
Stability of huAb45 monoclonal antibody. (A) The measurement of the binding of huAb45 to NGF after light exposure by ELISA. (B) The measurement of the binding of huAb45 to NGF after being heated at 60 °C and 70 °C for 1 h. (C) The neutralizing activities of huAb45 after being heated at 60 °C and 70 °C for 1 h. Data shown represent *n* = 3. (D) The purity of huAb45 after being heated at 60 °C and 70 °C for 1 h analyzed by SEC‐HPLC. Error bars represent the SD.

ELISA was used to measure the binding ability of huAb45 after being treated by light. HuAb45 displayed excellent binding affinities after light exposure, with an EC_50_ of 0.01086 and 0.01063 nm before and after light exposure, respectively (Fig. 4A). Then huAb45 was heated at 60 °C or 70 °C for 1 h, and it showed almost the same binding and blocking abilities after being heated at 60 °C for 1 h, but these abilities decreased after being heated at 70 °C for 1 h (Fig. 4B,C). Finally, the purity of huAb45 was analyzed by SEC‐HPLC after being heated, and huAb45 exhibited great thermostability at 60 °C, but showed a decrease at 70 °C (Fig. 4D).

### HuAb45 and tanezumab bind to different epitopes

**Fig. 5:**
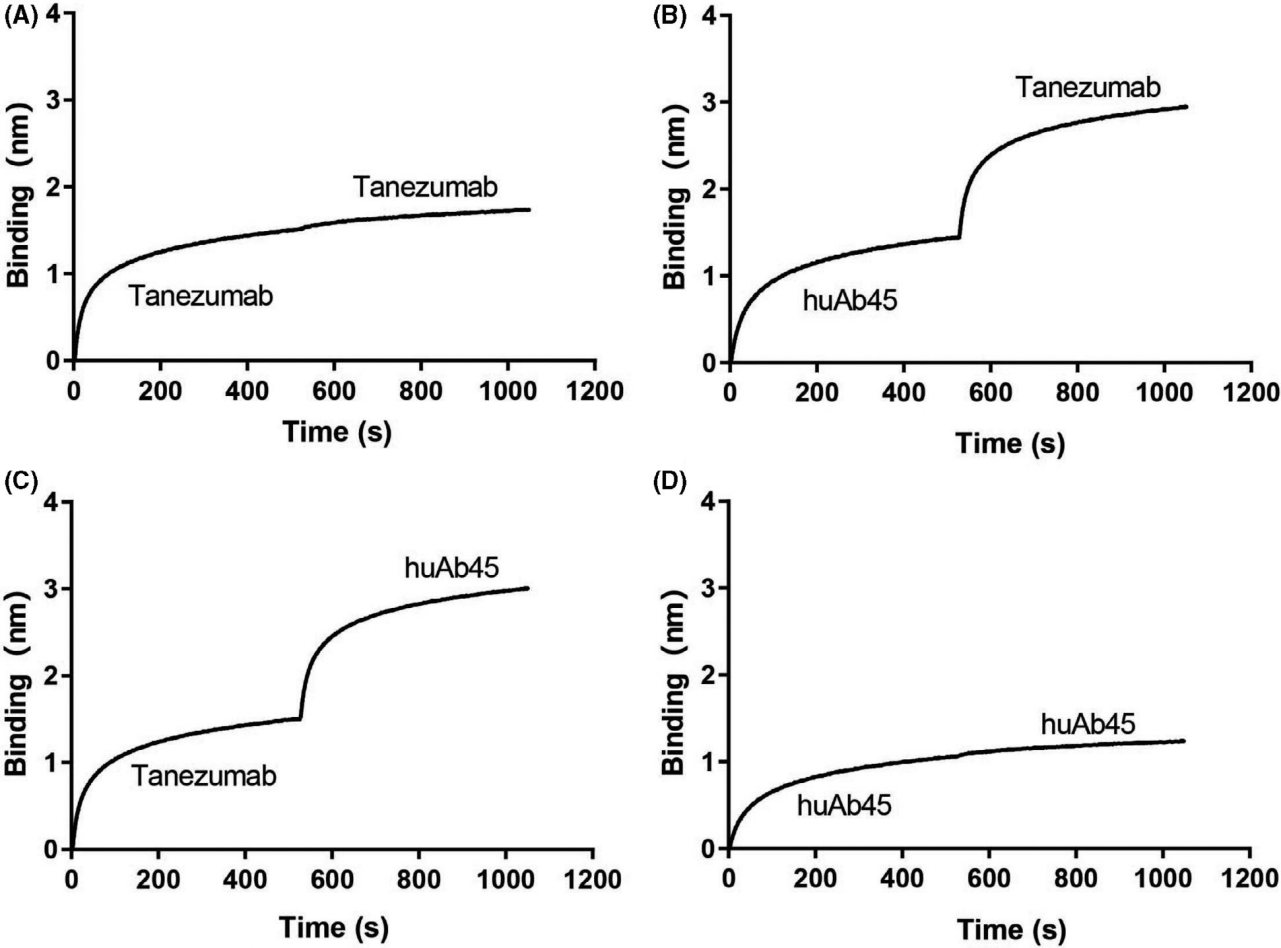
Octet RED96 Binning. Tanezumab and huAb45 were probed for binding against the same antigen (human NGF‐6xHis). In this typical binning assay, antibodies were loaded onto protein A sensors and blocked with a human IgG2 antibody, and the sensors were exposed to human NGF‐6xHis followed by the second antibody. (A) Tanezumab (100 nm) was loaded onto sensors and exposed to solutions of 100 nm NGF‐6xHis followed by 100 nm tanezumab. (B) HuAb45 (100 nm) was loaded onto sensors and exposed to solutions of 100 nm NGF‐6xHis followed by 100 nm tanezumab. (C) Tanezumab (100 nm) was loaded onto sensors and exposed to solutions of 100 nm NGF‐6xHis followed by 100 nm huAb45. (D) HuAb45 (100 nm) was loaded onto sensors and exposed to solutions of 100 nm NGF‐6xHis followed by 100 nm huAb45.

In order to test whether huAb45 and tanezumab bind to the same or different epitopes on NGF, the two antibodies were loaded in different orders. When tanezumab was loaded as the first antibody, and huAb45 was defined as the second, a significant binding signal could be observed in the association step (Fig. 5C). Conversely, when huAb45 was loaded as the first antibody, and tanezumab was defined as the second, a significant binding signal could also be observed in the association step (Fig. 5B). Whereas, when one antibody was defined as both the first and second antibody, no additional signal was observed. Thus, we concluded that huAb45 and tanezumab bind to different epitopes on NGF (Fig. 5A,D).

### HuAb45 showed analgesic effects in a mouse arthritis model

**Fig. 6:**
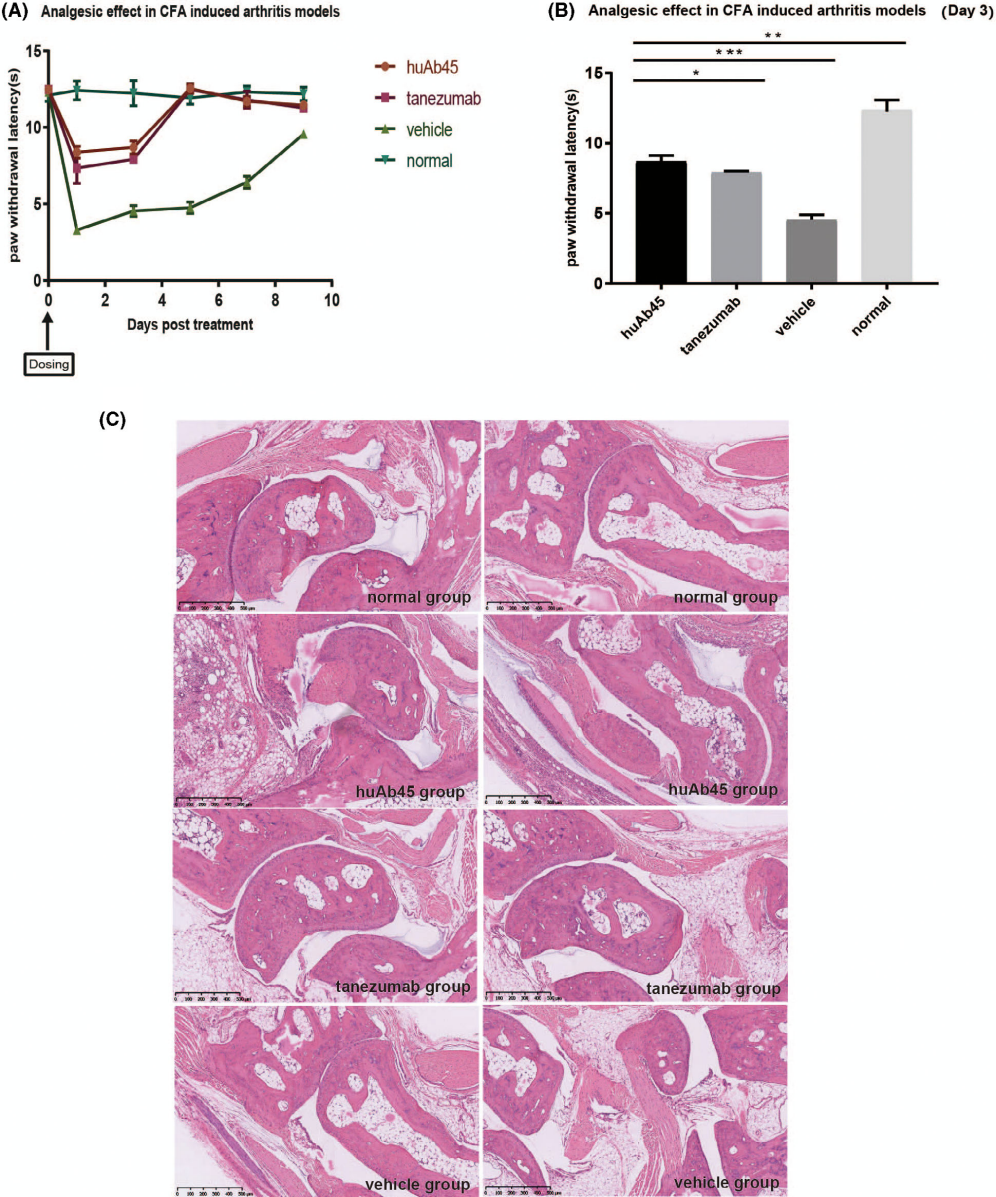
Analgesic effects of huAb45 on CFA‐induced mouse arthritis models. (A) Analgesic effects. C57BL/6 mice were sub‐plantar injected with huAb45 and tanezumab at the dose of 2.5 mg·kg^−1^, respectively on D0. (B) The analgesic latency of huAb45 was evaluated by comparison of 50% g threshold to each group on D3. (C) HE staining of the mice metatarsophalangeal joints. Data shown represent *n* = 6 (Dunnett’s multiple comparisons test). Error bars represent SD; *P < 0.05; **P < 0.01; ***P < 0.001. Scale bar, 100 μm.

In the C57BL/6 mouse arthritis model induced by complete Freud's adjuvant (CFA), huAb45 exhibited better analgesic effects than tanezumab (Fig. 6A,B), and it did not exacerbate the deterioration of inflammation or tissue damage (Fig. 6C–J) as suggested by the Hematoxylin and eosin (HE) staining performed on the inflamed tissues, which showed that huAb45 has analgesic effects on a mouse arthritis model.

### HuAb45 exhibited analgesic activity in a mouse model of diabetic neuralgia

**Fig. 7:**
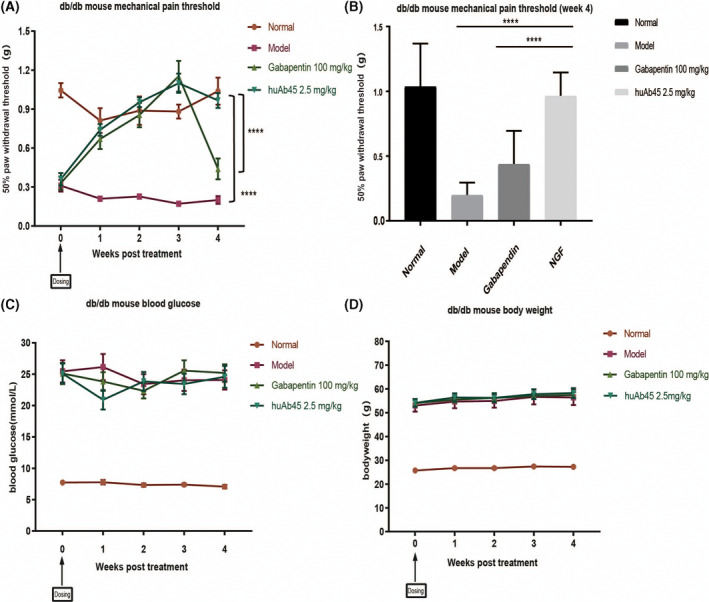
Analgesic effects of huAb45 on db/db mouse model of diabetic neuralgia. (A) HuAb45 treatment efficacy on db/db mice at the dose of 2.5 mg·kg^−1^ was analyzed by the von Frey method. (B) The comparison of 50% g threshold among groups at the 4th week (Dunnett’s multiple comparisons test). (C) Blood glucose changes of mice. (D) Changes in body weight changes of mice. All error bars indicate: SEM (*n* = 10). Error bars represent the SD; ****P < 0.0001.

The analgesic ability of huAb45 was validated in the db/db mouse model of diabetic neuralgia. HuAb45 showed analgesic effects similar to that of gabapentin in the first 3 weeks of the experiment, but surpassed the latter starting from the 4th week (Fig. 7A,B). During the experiment, huAb45 did not induce noticeable changes in body weight or blood glucose of the mice (Fig. 7C,D).

## Discussion

There is an increasing demand for medications with better therapeutic efficacy and fewer side effects for diabetic neuralgia [24, 25]. This study aims to generate an mAb that can be potentially applied to the clinical management of diabetic neuralgia.

A neutralizing NGF antibody was first discovered from hybridomas and humanized to huAb45, and then its binding and neutralizing abilities were evaluated at the molecular, cellular, and animal model levels. This molecule demonstrated high purity and stability, as well as the capacity to efficiently block the interaction between NGF and it high‐affinity receptor TrkA. More important, huAb45 binds to an antigen epitope different from tanezumab (Fig. 5B,C). In the CFA‐induced arthritis model, huAb45 showed better analgesic efficacy than tanezumab (Fig. 6B). In the db/db mouse model of diabetic neuralgia, huAb45 displayed more sustainability than gabapentin (Fig. 7B). These findings have shown that we were successful in generating a novel NGF mAb that may bring long‐term clinical benefits to patients with diabetic neuralgia.

Tanezumab, an NGF mAb jointly promoted by Pfizer and Eli Lilly, has demonstrated impressive analgesic efficacy in multiple clinical trials [26, 27]. However, during the clinical trials of OA, some patients acquired RPOA after the 80 weeks of antibody treatment [28]. The pathological mechanism of RPOA caused by tanezumab has long been debated, but it seemed that this side effect was limited mostly to OA patients. It is worth noting that when tanezumab was used together with nonsteroidal antiinflammatory drugs (NSAIDs) [29], patients were significantly more likely to develop RPOA, which serves as a reminder of excluding OA patients and other patients who take NSAIDs in the development of NGF antibody therapeutics [30].

The application of NGF antibodies is a promising strategy for the treatment of diabetic neuralgia. First, there is currently no effective pain‐managing medicines and tanezumab has shown remarkable efficacy in a Phase II clinical trial for diabetic neuralgia. Second, diabetic neuralgia is a non‐OA indication and NSAIDs are not typically used in diabetic patients. Therefore, the risk of causing RPOA in diabetic neuralgia by NGF antibodies is really low [31]. In the clinical trials of tanezumab, it was administered every 8 weeks, which increases the interval between dosing and makes the medicine more convenient to use. Furthermore, NGF antibodies are not expected to be prone to drug resistance, which may lead to sustained effects on diabetic neuralgia.

Overall, we generated huAb45, a NGF‐neutralizing antibody binding to a novel epitope. It has been demonstrated that huAb45 successfully relieved the pain in an animal model of diabetic neuralgia. More important, its analgesic effects in diabetic neuralgia and arthralgia experiments were superior to that of positive controls. This mAb is expected to substantially minimize the pain caused by diabetes and provide patients with benefit of convenient administration.

## Conflict of Interest

Xingchen Dong, Yili Chen, Huanhuan Li, Jie Tan, Ganjun Chen, Santai Li, and Zuobin Xie received stipends from Shanghai Mabstone Biotechnology, Co., Ltd.

## Data Accessibility

Data are available from the corresponding author upon reasonable request.

## Author contributions

Conceptualization, Y.L. C. and C.W.; methodology, Y.‐L. C.; validation, X. D., G. L., Y. C, and H. L.; formal analysis, Y.C., X. D., and C.W.; investigation, Y.‐L. C. and C.W.; resources, Y. L. C., C.W..; data curation, S, L. and X. D.; writing—original draft preparation, X. D.; writing—review and editing, Y.‐L. C., X. D and C.W.; visualization, X.D., M.P., and Z.X.; project administration, Y.‐L. C.; funding acquisition, C.W. All authors have read and agreed to the published version of the article.
